# Multi-Sensor Extrinsic Calibration Using an Extended Set of Pairwise Geometric Transformations

**DOI:** 10.3390/s20236717

**Published:** 2020-11-24

**Authors:** Vitor Santos, Daniela Rato, Paulo Dias, Miguel Oliveira

**Affiliations:** 1 Department of Mechanical Engineering(DEM), Institute of Electronics and Informatics Engineering of Aveiro (IEETA), University of Aveiro, 3810-193 Aveiro, Portugal; danielarato@ua.pt (D.R.); mriem@ua.pt (M.O.); 2 Departament of Electronics, Telecommunications and Informatics (DETI), Institute of Electronics and Informatics Engineering of Aveiro (IEETA), University of Aveiro, 3810-193 Aveiro, Portugal; paulo.dias@ua.pt

**Keywords:** calibration, multimodality, extrinsic parameters, point cloud, transformation path, Chauvenet criterion, singular value decomposition, ATLASCAR

## Abstract

Systems composed of multiple sensors for exteroceptive perception are becoming increasingly common, such as mobile robots or highly monitored spaces. However, to combine and fuse those sensors to create a larger and more robust representation of the perceived scene, the sensors need to be properly registered among them, that is, all relative geometric transformations must be known. This calibration procedure is challenging as, traditionally, human intervention is required in variate extents. This paper proposes a nearly automatic method where the best set of geometric transformations among any number of sensors is obtained by processing and combining the individual pairwise transformations obtained from an experimental method. Besides eliminating some experimental outliers with a standard criterion, the method exploits the possibility of obtaining better geometric transformations between all pairs of sensors by combining them within some restrictions to obtain a more precise transformation, and thus a better calibration. Although other data sources are possible, in this approach, 3D point clouds are obtained by each sensor, which correspond to the successive centers of a moving ball its field of view. The method can be applied to any sensors able to detect the ball and the 3D position of its center, namely, LIDARs, mono cameras (visual or infrared), stereo cameras, and TOF cameras. Results demonstrate that calibration is improved when compared to methods in previous works that do not address the outliers problem and, depending on the context, as explained in the results section, the multi-pairwise technique can be used in two different methodologies to reduce uncertainty in the calibration process.

## 1. Introduction

Modern robots count on a wealth of sensors for many essential operations that need perception such as representation, obstacle avoidance, planning, guidance, localization, and most of the tasks generically related to navigation and safety. Sensors now cover a wide scope of principles and modalities; therefore, it is no longer unexpected to see altogether monocular (both visual and infrared) and stereo cameras along with structured light-based or TOF 3D cameras and LiDAR (2D and 3D) mounted on some more complex systems like autonomous cars.

One of the first challenges before using such complex robotic systems is to calibrate all these sensors so their data or deduced conclusions can be merged and reported to a common coordinate frame for the algorithms to apply on a rich set of sensor data. This redundancy is necessary to give robustness and also to cover potential variations of data rate that may ultimately jeopardize single sensor or single modality based perception.

Reporting all sensors to a common frame can be simply stated as having the knowledge of where (translation and orientation) is each sensor coordinate frame relatively a common reference, normally one associated or easily related to the external world being perceived. These parameters are known, for each sensor, as its extrinsic parameters.

Numerous works exist to calculate the extrinsic parameters of cameras or image based or image reducible sensors. External devices such as chessboards, charuco boards, or others are used to create a known real-world pattern of points or geometric feature which are easily traceable on the respective images. Using these real-world references and the knowledge of the projection mechanisms (through the so-called intrinsic parameters of each device), it is possible to deduce the extrinsic parameters of a sensor in relation to the target being scanned.

In theory, having such a marker (chessboard or similar) shown to all sensors at once would allow for the computation of the extrinsic parameters for all sensors and the problem would be solved: that marker (chessboard or similar) should only be placed in a known and interesting or convenient position to the robot and all sensors would be easily localized relatively to a common reference frame.

The problem is that not all sensors may be able to perceive the target in ideal conditions and not all sensors are able to detect the target with the same representation, or may not even detect the target at all. Additionally, there are uncertainties in the process.

**Figure 1:**
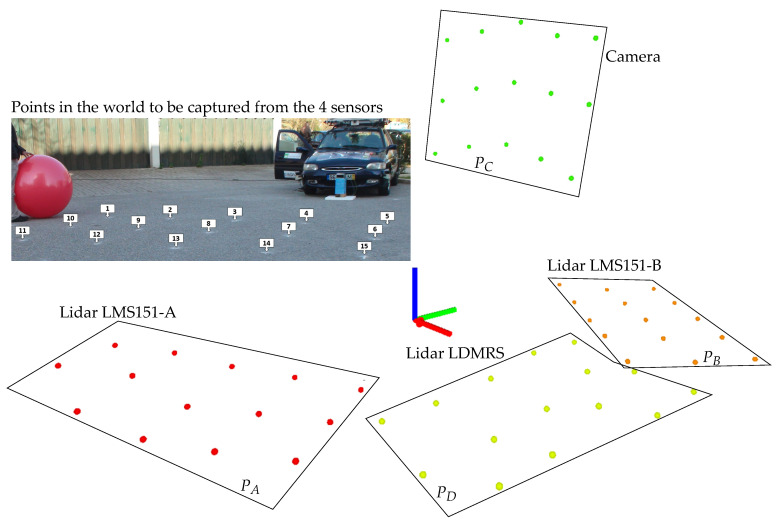
Example of four unregistered point clouds in ATLASCAR1 set-up (adapted from [1]).

An illustration of a real historical setup that will serve as base for developments ahead is shown in Figure 1 where four sensors (three LiDARS and one camera) generate a point cloud each, all obtained from the same temporal scenario (the center of a ball in several positions) but on their own coordinate frames. It is clear that the relative positions of the sensors must be determined in order to fuse or combine the four point clouds.

The main contribution of this paper is a technique to perform the extrinsic calibration of multiple sensors by eliminating outliers in the experimental process and by combining individual pairwise transformations. It improves a previously developed technique based on pairwise geometric transformations obtained from different point clouds (one for each sensor) generated with the successive center points of a moving ball.

The paper is divided in the following main sections; the related work, the proposed approach that includes the main algorithms described in detail, results from simulated and real data experiments, and final conclusions and future perspectives.

## 2. Related Work

Extrinsic calibration is a basic requirement in multi-sensor platforms where data need to be represented in a common reference frame for data fusion and subsequent analysis. This calibration procedure estimates the transformation between all sensors to align all datasets in the same coordinate system. Most calibration procedures are based in calibration patterns to ensure a robust and accurate detection of points/objects by all sensors. Some examples of calibration patterns are chessboards [2,3,4], fiducial markers [3,5,6], spherical objects [1,7,8,9], or cylindrical objects [10].

Many calibration systems are described in the literature; however, there is no general solution multiple sensor calibration. Pairwise calibrations between sensors are often used due to their simplicity, as the calibration step does not require a global optimization. This pairwise approach must consider all possible combinations between sensor modalities in the pair. These sensor combinations have been addressed in the literature: RGB to RGB camera calibration [1,11,12,13,14,15], RGB to depth camera (RGB-D cameras) calibration [9,16,17,18,19,20], camera to 2D LIDAR [1,4,19,21,22,23,24,25], 2D LIDAR to 3D LIDAR [10], camera to 3D LIDAR [25,26,27], and camera to radar [28].

To adapt the pairwise approach to the case of complex robotic systems that contain many sensors of different modalities, several pairwise calibrations must be combined in a sequential transformation procedure based on graphs where one sensor successively calibrates with another and then relates to a another sensor. Another approach is to define one sensor as the reference and report all the remainder to it. In this case, the graph of transformations is a one-level pyramid with the reference sensor on top and all other sensors below. This methodology is the one adopted in [1] to calibrate all the sensors on-board the ATLASCAR autonomous vehicle [29] relatively to a reference sensor.

The problem of multi-sensor calibration can also be solved using simultaneous optimization, as in Liao et al. [30] that use a joint objective function to calibrate simultaneously three RGB cameras and a RGB-D camera with good results in the calibration accuracy. An approach to estimate simultaneously temporal offsets and spatial transformations is presented in [31]. This approach can be used for any set of sensors (for example, cameras and LIDAR), as its does not consider unique properties of specific sensors. It also does not require the usage of calibration patterns for the LIDAR, as the planes present in the scene are used for that purpose. Another relevant work occurs in [32] that proposes a calibration for the sensors onboard a PR2 robot. The process uses the sensor uncertainty and is based on bundle adjustment.

In [33], an optimization procedure is implemented which, in addition to estimate the poses of the sensors, also estimates the poses of the calibration patterns. This enables the definition of errors to be formulated using sensor to calibration pattern tandems, rather than the classic sensor to sensor pairwise combinations. As a result, the problem of the exploding number of sensor combinations is avoided, as the number of combinations do not explode with the increase in the number of sensors, which makes it possible to consider all available data during the optimization.

In most of the mentioned approaches, there is still the need for some sort of user interaction, or to provide first guess estimates for optimization based algorithms, which sometimes may be slow to converge, despite the fact that that slowness may not be relevant for a offline process, which is the case for many calibration procedures.

Overall, it appears pertinent to devise a solution of a nearly automatic mechanism with very little intervention of human operation, desirably with sleek performance. One solution, already exploited by other authors [1], is to use a simple target that must be easily detectable by all sensors, from a wide range of viewpoints and perspectives, be it based on images, range maps, or even simple 2D range profiles. Pereira et al. [1] used a large size ball that is easily detected by cameras and LiDARs (including TOF and structured light devices). Those works have been later extended [8] to monochromatic and infrared-based images using Deep Learning techniques to detect the ball in the image with greater robustness and accuracy. These solutions, however, besides still relying on pairwise approaches, do not address the problem of outliers, which this papers addresses by proposing an extended solution that combines the pairwise transformation and also automatically filters out outliers generated in the acquisition process.

The work clearly follows the line of the works in [1,8], but it integrates and extends the concepts to multi-pairwise procedures. Compared to existing works, the proposal that is going to be detailed in the paper has the following advantages; (i) it can be applied to any sensor that is capable of detecting the 3D position of a moving target (ball), and not only cameras; (ii) it can take all transformation paths into account or a subset of paths; (iii) it is faster than iterative methods with deterministic duration and computational cost; and (iv) in certain conditions (explained further), it allows to reduce calibration errors in a straightforward algorithmic process.

## 3. Proposed Approach

**Figure 2:**
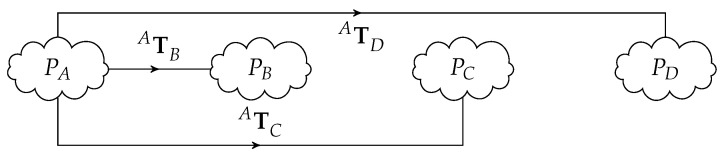
Example of four distinct point clouds showing the transformations of B, C, and D relative to frame A. See the set-up in Figure 1.

Figure 1 and its associated diagram in Figure 2 illustrate the acquisition of the same scene by four different and arbitrarily positioned sensors (A, B, C, D), generating four point clouds corresponding to several positions of a known object seen by all sensors at different locations in the scene like the center of a ball, for example ($P_{A},P_{B},P_{C},P_{D}$). For the sake of clarification, these point clouds are not actually the usual point clouds that express the geometry of a scene; they are instead a set of points that represent the time-lapsed position of a moving object in space, in this case the successive sparsely distributed positions of a ball center. However, theoretically, these two types of point clouds have similar formats and can be manipulated with common available tools.

Simple 3D data set matching, or more sophisticated registration techniques readily available in many libraries (Point Cloud Library (PCL) or open3D, for example), can be used to obtain the position of sensors B, C, and D relatively to sensor A, which is assumed to be the reference of the system: this is translated by the three geometric transformations ${}^{A}T_{B}^{}$, ${}^{A}T_{C}^{}$, ${}^{A}T_{D}^{}$. In summary, for a set of four sensors (four point clouds), three matching operations are performed, that is, take one sensor (A) as the reference, and determine the position of the remainder three in relation to it.

**Figure 3:**
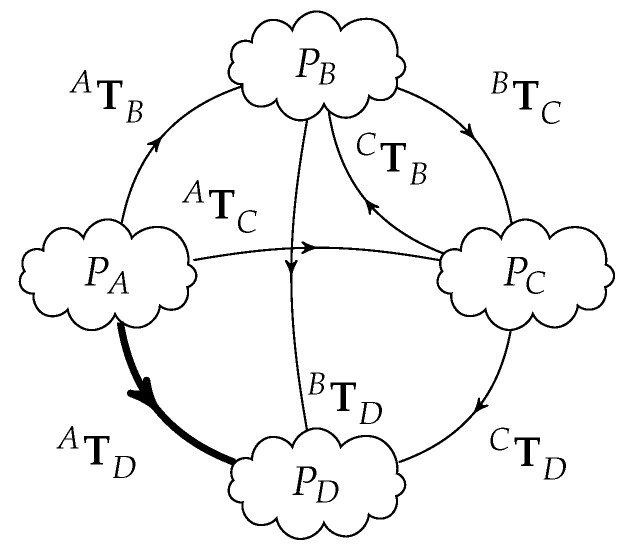
Rearrangement of example from Figure 2 of four distinct point clouds, but now showing all the paths for transformations from A to D, passing also through B and C.

However, there are other transformations that can be obtained among the remainder sensors using their own point clouds besides sensor A. Figure 3 rearranges the layout and shows all possible transformation paths to obtain ${}^{A}T_{D}^{}$; a similar reasoning can be done to obtain any of the other transformations in diverse representations of equivalent transformation paths.

We call a transformation path ${}^{X}T_{Y}^{}$, from frame *X* to frame *Y*, a sequence of geometric transformations derived from the transformation graph. An additional superscript *k* is used to distinguish different transformation paths between the same coordinate frames ${}^{X}T_{Y}^{k}$. For example, as shown in Figure 3, there are five transformation paths (different ways) to obtain ${}^{A}T_{D}^{}$, after ${}^{A}T_{B}^{}$, ${}^{B}T_{C}^{}$, ${}^{A}T_{C}^{}$, ${}^{C}T_{D}^{}$, ${}^{B}T_{D}^{}$, and ${}^{C}T_{B}^{}$, reminding, however, that ${}^{C}T_{B}^{}={\left({}^{B}T_{C}^{}\right)}^{-1}$:

1. the direct measurement of ${}^{A}T_{D}^{}$ from the data matching algorithm: ${}^{A}T_{D}^{0}={}^{A}T_{D}^{}$
2. ${}^{A}T_{D}^{1}={}^{A}T_{B}^{}{}^{B}T_{D}^{}$;
3. ${}^{A}T_{D}^{2}={}^{A}T_{C}^{}{}^{C}T_{D}^{}$;
4. ${}^{A}T_{D}^{3}={}^{A}T_{B}^{}{}^{B}T_{C}^{}{}^{C}T_{D}^{}$;
5. ${}^{A}T_{D}^{4}={}^{A}T_{C}^{}{}^{C}T_{B}^{}{}^{B}T_{D}^{}={}^{A}T_{C}^{}{\left({}^{B}T_{C}^{}\right)}^{-1}{}^{B}T_{D}^{}$

It is certain that these geometric transformations are independent because the point clouds were created from the point of view of different sensors. As an hypothetical situation: if, for example, the $P_{D}$ point cloud has poorer quality (due perhaps to more noisy acquisition settings) the ${}^{A}T_{D}^{}$ transformation would exhibit some larger uncertainties; so, combining this transformation with other transformations that involve other (expectantly) more precise point clouds will improve a final version of ${}^{A}T_{D}^{}$, but involving of course the $P_{D}$ point cloud. As described ahead, a better estimate of ${}^{A}T_{D}^{}$ can be obtained by the combination of part or all of the five listed results (${}^{A}T_{D}^{0}$,${}^{A}T_{D}^{1}$,${}^{A}T_{D}^{2}$,${}^{A}T_{D}^{3}$,${}^{A}T_{D}^{4}$). The 0-th transformation path (k=0) is the actual direct transformation from sensor *A* to *D* in the current example.

With these transformation paths it is possible to calculate a geometric transformation that is some type of combination of all the transformation paths, expectantly with a smaller uncertainty than the single original transformation.

To ease the interpretation of equations and algorithms, the following nomenclature convention is adopted to describe geometric transformations from sensor *i* to sensor *j*.

- ${}^{i}T_{j}^{}$—The real transformation (usually unknown)
- ${}^{i}{\tilde{T}}_{j}^{}$—The “measured” transformation (after the common localization of a unique object)
- ${}^{i}{\hat{T}}_{j}^{}$—The estimated (calculated, hopefully improved) transformation.
- ${}^{i}{\hat{T}}_{j}^{k}$—A derived transformation resulting from some transformation path ${}^{i}T_{j}^{k}$.

The estimated transformation is actually calculated using some sort of mean or combination of multiple derived transformations which are the result of their associated transformation paths. It is clear then, that the concept of transformation path (which results in derived transformations) is useful in a numeric approach because independent transformations can be obtained experimentally with, possibly, different levels of uncertainty, and their manipulation can provide an averaging or smoothing of those uncertainties. As stated earlier, a derived transformation (as one sample *k* of a larger set) is represented by ${}^{n}{\hat{T}}_{m}^{k}$, which means the k-th sample of the derived transformations from sensor *n* to *m*.

Each derived transformation, for example, between sensor 0 and sensor *n*, can be obtained in several ways, depending on how many steps the accumulated concatenation of transformations (transformation path) is done, like the following example.

(1) $$\begin{array}{cc} {}^{0}{\hat{T}}_{n}^{1} & ={}^{0}{\tilde{T}}_{1}^{}{}^{1}{\tilde{T}}_{n}^{}\\ {}^{0}{\hat{T}}_{n}^{2} & ={}^{0}{\tilde{T}}_{2}^{}{}^{2}{\tilde{T}}_{n}^{}\\ {}^{0}{\hat{T}}_{n}^{3} & ={}^{0}{\tilde{T}}_{3}^{}{}^{3}{\tilde{T}}_{n}^{}\\ \; & \cdots \\ {}^{0}{\hat{T}}_{n}^{k} & ={}^{0}{\tilde{T}}_{p}^{}{}^{p}{\tilde{T}}_{q}^{}\cdots {}^{m}{\tilde{T}}_{n}^{}. \end{array}$$

Those derived transformations can be combined (averaged) and compared, or even merged, with the actual measurement (${}^{0}{\tilde{T}}_{n}^{}$) to provide a result with more confidence than the actual measurement itself, and is given in the general case by

(2) $${}^{0}{\hat{T}}_{n}^{}=\mathrm{COMBINE}\left({}^{0}{\tilde{T}}_{n}^{},{}^{0}{\hat{T}}_{n}^{1},{}^{0}{\hat{T}}_{n}^{2},{}^{0}{\hat{T}}_{n}^{3},\cdots ,{}^{0}{\hat{T}}_{n}^{k}\right),$$

where COMBINE() represents the function to average, merge, weight, or otherwise combine samples of a transformation between two sensors, although originated from distinct transformation paths; from now on, the terms “average” and “combine” for geometric transformations will be used interchangeably. The algorithm for this multi-pairwise approach is detailed further in Section 3.2.

In this process, translations and rotations are expected to be combined separately and outliers are to be taken into account, as well as the confidence of each sample, should it be available or known, for example, based on the number of points present in each point cloud: assuming that, the more the points, the better is the estimation of the geometric transformation, which may be debatable, mainly because of poor detections or the presence of outliers.

Resuming to the example of Figure 3, there are $6=\frac{4\times 3}{2}$ direct feedforward transformations (pairwise independent relations) that can be obtained from the four measured point clouds. These six transformations have more information than only the three related to a single reference frame.

In summary, the idea is to define one sensor from the set of *N* sensors to be the reference (usually the one easier to place or locate in the overall reference frame of the world), naming it A, whichever it might be, and obtain the relative position of the remainder (N−1) sensors relatively the reference. Each of these (N−1) transformations is now to be obtained as an “averaging” of a number of separate geometric transformations that represent the same frame relations.

This implies that, whenever needed, matching operations have to be done both ways, like ${}^{C}T_{D}^{}$ and ${}^{D}T_{C}^{}$; however, as ${}^{D}T_{C}^{}={\left({}^{C}T_{D}^{}\right)}^{-1}$, in principle, inverting a matrix can be performed instead of a second point cloud matching; nonetheless, most of the times, point cloud matching algorithms, namely, when probabilistic approaches are used, can perform differently when source and target point clouds are swapped, and therefore the safest option is to calculate both and pick the best instead of simply inverting matrices.

For a set of *N* sensors, a global overview of the operations can be summarized as follows.

- Acquire *N* point clouds of a reference object in several positions, one from each sensor;
- Perform at most N(N−1) point cloud matching operations or, assuming that ${}^{n}{\tilde{T}}_{0}^{}$ will not be used, perform $\left(N-1\right)+\left(N-1\right)\left(N-2\right)={\left(N-1\right)}^{2}$ point cloud matching operations;
  - Alternatively, perform only $\left(N-1\right)+\frac{\left(N-1)(N-2\right)}{2}=\frac{N(N-1)}{2}$ cloud matching operations, and $\frac{\left(N-1)(N-2\right)}{2}$ inversions of transformations (in reverse directions of the previous point). In this case, less than $\frac{N(N-1)}{2}$ inversions are necessary because no inverse transformations are made to the reference sensor.
- Each of the N−1 sensors has a set of transformation paths (connecting to the reference sensor) with different lengths (1, 2, 3, …) where the length of some transformation path $L\left({}^{X}T_{Y}\right)$ is the number of transformations that compose it, as enumerated next, where P(n,r) is the permutation (arrangements without repetition) of *n* elements taken in groups of *r* elements:
  - length 1: P(N−2,0)=(N−2)!/(N−2)!=1, one path;
  - length 2: P(N−2,1)=(N−2)!/(N−3)!=(N−2) paths;
  - length 3: P(N−2,2)=(N−2)!/(N−4)!=(N−2)(N−3) paths;
  - ⋯
  - length *r*: P(N−2,r−1) paths (for r<N);
  with a total number of paths $Q_{N}$ for each of the N−1 sensors given by:
  (3) $$Q_{N}=\sum_{r=1}^{N-1}P\left(N-2,r-1\right),$$
  where *N* is the number of sensors, *r* is the length of transformation path, and P means the mathematical permutation, as stated above.
- Perform (N−1) averaging operations of geometric transformations to obtain all ${}^{0}{\hat{T}}_{n}^{}$.

For the illustrated case of N=4, for each sensor relatively to the reference sensor, there is one transformation path with length 1, two paths with length 2, and two paths with length 3, yielding a total number of transformation paths given by (4−1)×(1+2+2)=15. Table 1 shows the number of transformation paths for all the possible path lengths in a set of several sensors (from 3 to 10).

The number of paths grows exponentially with the number of sensors. As an indication, 11 sensors imply a total of nearly a million transformation paths, and 20 sensors would generate more than $10^{16}$ paths. For those cases where many transformation paths exist, a solution can be to limit the number of transformation paths and not use all of them, as the principle applies independently of the number of the transformation paths to be “averaged”. More transformation paths should reduce the uncertainty but it is expected that after a certain number, that reduction may become negligible, and therefore no longer useful. This statement is a generalization of the concept of uncertainty propagation in an averaging process of *N* samples of some variable; if *N* is large, using N+1 samples is not expected to reduce much further the uncertainty of the averaged result.

Possible strategies to limit the number of transformation paths to combine, for each sensor, include the following.

1. Use minimum path lengths, but ensuring all combinations of that path length—this require path lengths of 2, but all of them are necessary to involve all sensors in the estimations of each ${}^{0}{\hat{T}}_{n}^{}$. This would require (N−2) transformation paths to be combined with ${}^{0}{\tilde{T}}_{n}^{}$ for each sensor.
2. Use a maximum path length which ensures that all sensors are involved as well, but no more than one path would be needed to cover all sensors. For each sensor, the estimation (averaging) would be done only with ${}^{0}{\tilde{T}}_{n}^{}$ and ${}^{0}{\hat{T}}_{n}^{k_{L}}$, where $k_{L}$ is the index that corresponds to one of the maximum path lengths.
3. Use a minimum number of transformation paths, but at least as large as the number of sensors in the set and, again, ensuring that all sensors are involved. Resorting to Table 1, this would require a path length of 3 (or just 2 for three sensors). The number of paths of length 3 to average for each sensor would be *N*, from all the possible available in the 4−th column of Table 1.

These possibilities are presented as heuristic alternatives to the usage of all transformation paths that, besides not being sure to be needed, would be impractical to apply for large values of *N*. However, values of *N* up to 6 are absolutely reasonable to use all transformation paths and keep the fast performance. There is no formal demonstration of which is the choice that produces best results, and a compromise solution with a large applicability would be to use all the transformation paths of length up to 3. Even large number of sensors would require only some hundreds of transformation paths. In this approach, it is assumed that it is always possible to establish transformation path between any pair of sensor with any viable path lengths. In case that turns out impossible (not all sensors share enough overlap of the field of view), a smaller number of paths has to be used.

### 3.1. Pairwise Matching Algorithm for Arrays of Points

To calculate the pairwise best fit transformation [R|t] between two sets of points *A* and *B*, the classical technique based on the work in [34] is used. These sets of points are sorted and there is a one-to-one correspondence between the points, but the expression “point cloud” will be used instinctively as “set of points”. The point clouds are first relocated around the origin (by subtracting them their respective centroids), and Singular Value Decomposition (SVD) is applied to the 3×3 covariance matrix of the coordinates of points (in the format 3×1), as expressed with Equations (4) and (5):

(4) $$A_{C}=A-centroid_{A}\;\mathrm{and}\;B_{C}=B-centroid_{B};$$

(5) $$H=A_{C}\;\cdot \;B_{C}^{⊺}\;\mathrm{and}\;\left[\begin{array}{ccc} U & \Sigma & V \end{array}\right]=\mathrm{SVD}\left(H\right).$$

The rotation matrix and the translation vector between the point clouds are consequently calculated using the expressions in (6):

(6) $$R=V\;\cdot \;U^{⊺}\;\mathrm{and}\;t=centroid_{B}-R\;\cdot \;centroid_{A}.$$

### 3.2. The Multi Pairwise Algorithm

**Figure 4:**
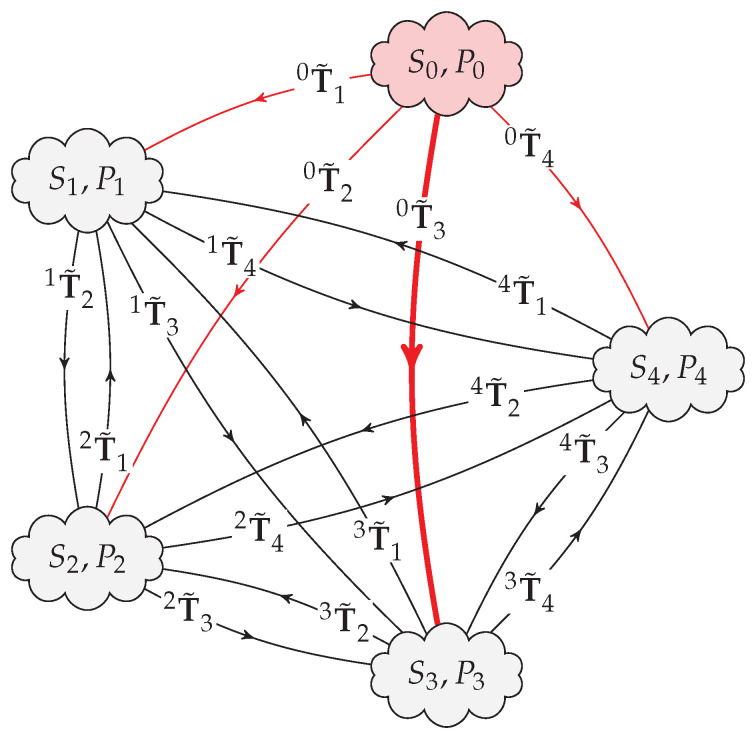
Example of five distinct sensors and their point clouds where all transformation paths from $S_{0}$ to $S_{3}$, passing also through $S_{1}$, $S_{2}$, and $S_{4}$, can be established. In the figure, transformations from $S_{0}$ to the other sensors are actually the ones who require improved estimates based on the several transformation paths and associated derived transformations, that is, calculate ${}^{0}{\hat{T}}_{n}^{}$ using the several ${}^{j}{\tilde{T}}_{i}^{}$ present in the diagram and obtained experimentally. Both ${}^{i}{\tilde{T}}_{j}^{}$ and ${}^{j}{\tilde{T}}_{i}^{}$ may be obtained using the same registration technique or, to save time, just by performing algebraic inversion: ${}^{i}{\tilde{T}}_{j}^{}={\left({}^{j}{\tilde{T}}_{i}^{}\right)}^{-1}$.

For the sake of simplicity, it is considered from now on that the sensors and their point clouds are numbered starting on zero ($S_{0}$ with $P_{0}$, $S_{1}$ with $P_{1}$, etc.), and that the reference sensor is sensor zero, as illustrated in Figure 4, where, as an example, what is sought is the position of sensor 3 ($S_{3}$) relatively to sensor 0 ($S_{0}$), that is, to obtain ${}^{0}{\hat{T}}_{3}^{}$. In the figure, reverse transformations to $S_{0}$ are not shown because they are not to be used in practice when calculating the derived transformations, that is, ${}^{n}{\tilde{T}}_{0}^{}$ will not be part of any transformation path.

The major steps of the algorithm were given earlier, but they can be detailed more specifically as follows.

- Define one sensor as the reference, $S_{0}$, to which all other sensors will be reported/located.
- The actual calibration procedure is to obtain the list of N−1 geometric transformations ${}^{0}{\hat{T}}_{n}^{}$ for n∈{1,2,⋯,N−1}.
- Acquire experimentally *N* point clouds $P_{n}$ from the *N* sensors.
- As the reference sensor is not to be part of any transformation path because it is never the destination of any transformation, perform $\left(N-1\right)+\left(N-1\right)\left(N-2\right)={\left(N-1\right)}^{2}$ point cloud matching operations, that is, obtain all pairs of geometric transformations ${}^{n}{\tilde{T}}_{m}^{}$ between sensor *n* and sensor *m*, where n,m∈{0,1,⋯,N−1} and m∉{0,n}. There is a partial alternative to this step described earlier but it is omitted here to keep the procedure shorter.
- Define which strategy is adopted to establish the set of transformation paths to use in the average calculation. As an example, variant 1 from the list presented earlier is chosen, meaning to pick all transformation paths with length 2, which is the minimum length, as proposed.
- For each sensor, perform the COMBINE calculation of the results obtained in the previous step.

In terms of pseudocode, the main procedure could be presented as shown in Algorithm 1.
**Algorithm 1:** Multi-pairwise sensor calibration
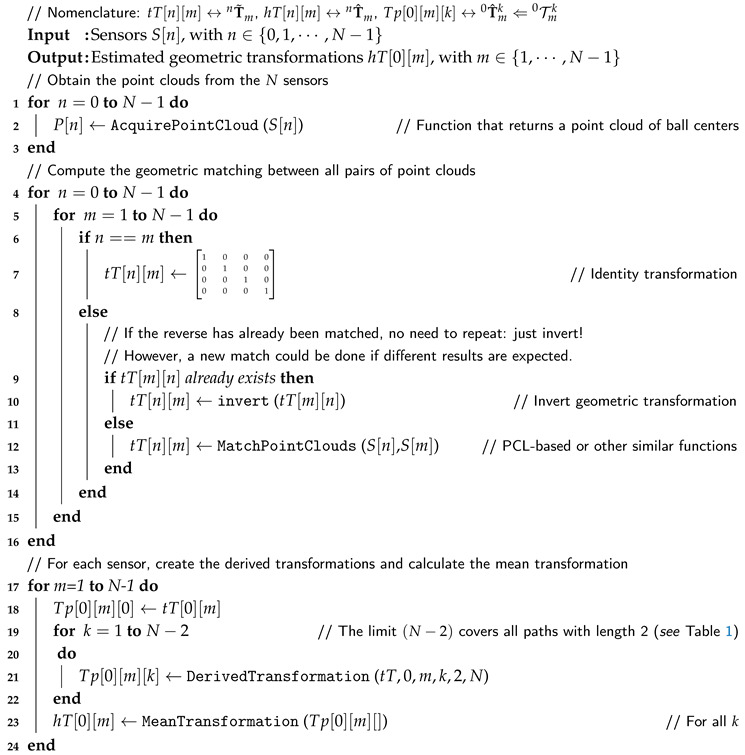


When ranging the transformation path number from k=0 to some limit $k=k_{L}$, an order is expected, where k=0 corresponds to a path with a single transformation (${}^{n}T_{m}^{0}={}^{n}{\hat{T}}_{m}^{}$) and the following values of *k* correspond to an increasing amount of accumulated transformations. For example, when N=5, for each of the four sensors relatively to the reference sensor, the following number of path lengths correspond to the indicated values of *k*:

$$\begin{array}{ccccc} \mathrm{Path}\;\mathrm{lengths}\;\mathrm{for}\;N=5\to & 1 & 2 & 3 & 4\\ \mathrm{Number}\;\mathrm{of}\;\mathrm{paths}\to & 1 & 3 & 6 & 6\\ \mathrm{Values}\;\mathrm{of}\;\mathrm{index}\;k\to & 0 & 1,2,3 & 4,5,6,7,8,9 & 10,11,12,13,14,15 \end{array}$$

Two of the most relevant functions from Algorithm 1 are DerivedTransformations() and MeanTransformation(). The latter can have several formulations as described further (with the SVD approach being the most straightforward to implement), but the first needs a little more explanation. The function requires the indices of the transformations from *n* to *m* of length *L* for path *k*, and use those indices to compose (post-multiply) the respective transformations tT[i][j]. The function and associate parameters are stated as follows.

Function *T* = DerivedTransformation(tT,n,m,k,L,N)

*T*  The return value: the derived transformation path Tp[0][m][0]
tT Array of transformation matrices for all pairs of sensors
*n*  Starting sensor (usually 0, but could be extended to be any)
*m*  Ending sensor (any, except sensor 0, but could be extended)
*k*  Number of the transformation path
*L*  Length of the transformation paths to use
*N*  Total number of sensors in the problem

According to what was stated earlier, for this call, the parameters *k* and *L* are redundant, but the function can be prepared for both approaches in the calling: if *k* is valid (k≥0) it has priority over *L* (which is then ignored or potentially used to confirm that there is no discrepancy between the desired *k* and the corresponding *L*); on the other hand (k<0, which is an invalid index), the first path of length *L* could be used and the appropriate value of *k* is assumed to perform the operation. For example, in the previous case, for N=5, the following call *T*=DerivedTransformation(tT,0,4,7,2,5) could trigger an alert because the value used for *k* (=7) corresponds to a path length of L=3 and not 2 as stated in the call. Still, as the path length of 3 remains compatible with the indicated number of sensors (=5), the calculation could be done, and the proper transformation for k=7, L=3, and N=5 would be returned.

To enable all this checking, the function must be able to assess the entire set of values for *k* for a given number of sensors *N* and for each path length *L*. That is given generically as shown next, where the number of path lengths ranges from 1 to N−1 and *k* is indeed function of *N* and *L*:

$$\begin{array}{cccccc} L & 1 & 2 & 3 & \cdots & N-1\\ & & 1 & (N-2)+1 & \cdots & \prod_{r=2}^{L-1}\left(N-r\right)+1\\ & & 2 & (N-2)+2 & \cdots & \prod_{r=2}^{L-1}\left(N-r\right)+2\\ k(N,L) & 0 & & & & \\ & & \vdots & \vdots & \vdots & \vdots \\ & & \left(N-2\right) & \left(N-2)+(N-2)(N-3\right) & \cdots & \prod_{r=2}^{L-1}\left(N-r\right)+\prod_{r=2}^{L}\left(N-r\right) \end{array}$$

In a more compact form, the previous statements can be summarized as

(7) $$k\left(N,L\right)\in \left\{K_{min},\cdots ,K_{max}\right\}=\left\{\prod_{r=2}^{L-1}\left(N-r\right)+1,\cdots ,\prod_{r=2}^{L-1}\left(N-r\right)+\prod_{r=2}^{L}\left(N-r\right)\right\},$$

knowing, of course, that these expressions are applicable for N≥3 and L<N, as is also verifiable in Table 1. Curiously, and it is simple to demonstrate, the following also holds,

(8) $$K_{max}=\left(K_{min}-1\right)\left(N-L+1\right).$$

To perform the computation of the k-th derived transformation (result of its correspondent transformation path), the function DerivedTransformation() needs the list of all permutations (arrangements) of (N−2) sensors taken in groups of *L*, but only those that end in the target sensor.

For example, five sensors taken in groups of 2, because path lengths of 2 are required, and excluding sensor 0—the reference, yield the following ordered permutations, (1 2), (1 3), (1 4), (2 1), (2 3), (2 4), (3 1), (3 2), (3 4), (4 1), (4 2), (4 3), however, when the target sensor to calibrate relatively sensor 0 is defined (and using the example of sensor 3), only the sequences that end in 3 are desired for further calculation: (1 3), (2 3), (4 3), that is, all the permutations of sensors {1,2,4} in groups of 1 (1=2−1=L−1) are needed. Therefore, the numbers of sensors in sequence whose transformations are to be obtained and further combined are 0⊳Perm({1,2,4},1)⊳3, where the symbol ⊳ denotes a transformation between the associated pair of sensors or, more explicitly

- $k=1\Rightarrow \left\{S_{0}⊳S_{1}⊳S_{3}\right\}\Rightarrow {}^{0}{\hat{T}}_{3}^{1}={}^{0}{\tilde{T}}_{1}^{}{}^{1}{\tilde{T}}_{3}^{}$
- $k=2\Rightarrow \left\{S_{0}⊳S_{2}⊳S_{3}\right\}\Rightarrow {}^{0}{\hat{T}}_{3}^{2}={}^{0}{\tilde{T}}_{2}^{}{}^{2}{\tilde{T}}_{3}^{}$
- $k=3\Rightarrow \left\{S_{0}⊳S_{4}⊳S_{3}\right\}\Rightarrow {}^{0}{\hat{T}}_{3}^{3}={}^{0}{\tilde{T}}_{4}^{}{}^{4}{\tilde{T}}_{3}^{}$

As another example, if N=6 and L=3 starting in $S_{0}$ and ending on $S_{4}$ would result in the following 12 sensor sequences to use 0⊳Perm({1,2,3,5},(3−1))⊳4 or, in expanded form (1 2 4), (1 3 4), (1 5 4), (2 1 4), (2 3 4), (2 5 4), (3 1 4), (3 2 4), (3 5 4), (5 1 4), (5 2 4), (5 3 4), still assuming, of course, that sensor 0 starts all sequences.

In conclusion, when function DerivedTransformation(tT,n,m,k,L,N) is called, for the sake and application in this paper, it expects the following integers and limits,

(9) n=0,N>2,0<L<N,0<m<N

and tT is an array with all pairs of geometric transformations previously calculated in Algorithm 1. The relevant code of the function is described in Algorithm 2.

However, if the length of the transformation paths is to be restricted to L=2 (as proposed earlier for the chosen solution to limit the number of operations), the procedures are simpler and Algorithm 2 can be simplified and proposed as in Algorithm 3.

Hence, the function GetPathPairSequences(*N*,*m*) from Algorithm 3 is much simpler than its general variant GetSensorPathSequences(*N*,*L*,*m*) from Algorithm 2 and provides the following sequences, being m>1 and N>2:

- 0⊳1⊳m
- 0⊳⋯⊳m
- 0⊳m−1⊳m
- 0⊳m+1⊳m
- 0⊳⋯⊳m
- 0⊳N−1⊳m

As mentioned earlier, the reference sensor is named zero and it is always the start of the transformation paths for any given target sensor. Should the reference sensor be another, a renaming of the sensors would be done to establish the new reference sensor, that is, the new sensor zero. Despite that possibility, the methodology remains unchanged, only the sensor numbering is affected.
**Algorithm 2:** Function to perform the computation of derived transformations
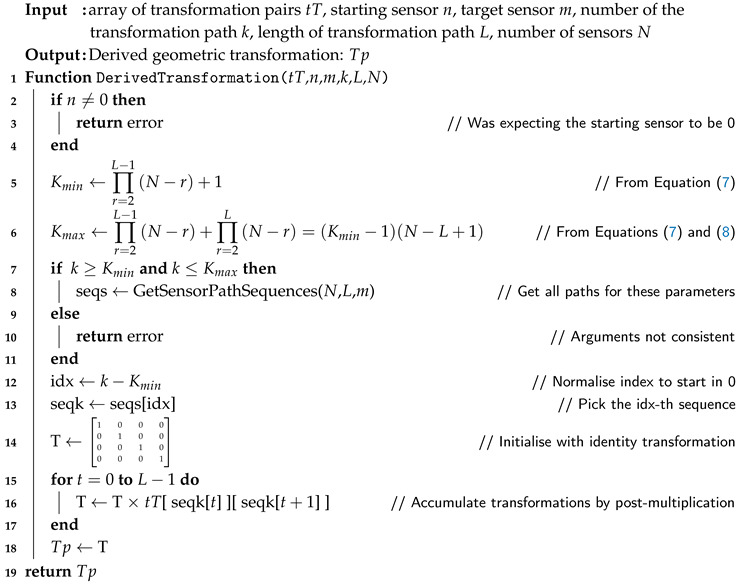
**Algorithm 3:** Simplified function to compute derived transformations with L=2
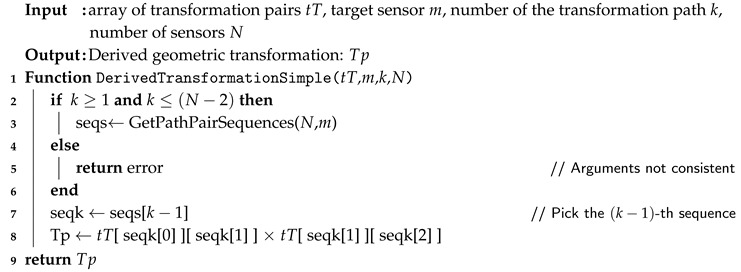


### 3.3. Combination of Geometric Transformations

Combining or averaging a set of transformations can be considered as averaging translations and rotation angles. Therefore, for a set of *K* transformation matrices given by ${}^{}T_{i}^{}=\left[\begin{array}{c} {}^{}R_{i}^{}\;|\;t_{i} \end{array}\right]$, the average transformation matrix has a translation vector which is given by $\hat{t}=\frac{1}{K}\sum_{i}^{K}t_{i}$ and a rotation matrix ${}^{}{\hat{R}}_{}^{}$ obtained by an operation of “averaging” the various ${}^{}R_{i}^{}$. A few approaches can be considered for this operation of merging the various ${}^{}R_{i}^{}$: quaternions, Euler angles, and single value decomposition (SVD) are the most likely candidates. Quaternions and Euler angle-based approaches both require a conversion between representations, but SVD does not. On the other hand, the Euler angles technique allows a true mean value calculation and also a weighted mean (in case it is necessary), making that approach very versatile in this context. Adopting the RPY version of Euler angles (but actually any triple of Euler angles would do), that formulates like this

(10) $$\begin{array}{cc} M & =\mathrm{RPY}(\phi ,\theta ,\psi )=\mathrm{rotz}(\phi )\times \mathrm{roty}(\theta )\times \mathrm{rotx}(\psi )\\ \; & =\left[\begin{array}{ccc} C\phi \;C\theta & C\phi \;S\psi \;S\theta -C\psi \;S\phi & S\phi \;S\psi +C\phi \;C\psi \;S\theta \\ C\theta \;S\phi & C\phi \;C\psi +S\phi \;S\psi \;S\theta & C\psi \;S\phi \;S\theta -C\phi \;S\psi \\ -S\theta & C\theta \;S\psi & C\psi \;C\theta \end{array}\right]=\left[\begin{array}{ccc} r_{11} & r_{12} & r_{13}\\ r_{21} & r_{22} & r_{23}\\ r_{31} & r_{32} & r_{33} \end{array}\right], \end{array}$$

and being $\theta \in ]-\pi /2,+\pi /2[$, then, the correspondent Euler angles can be expressed as

(11) $$\left[\begin{array}{c} \phi \\ \theta \\ \psi \end{array}\right]=E\left(M\right)=\left[\begin{array}{c} \arctan\left(r_{21},r_{11}\right)\\ \arctan\left(-r_{31},\sqrt{r_{32}^{2}+r_{33}^{2}}\right)\\ \arctan\left(r_{32},r_{33}\right) \end{array}\right].$$

If θ=±π/2 ($r_{11}=r_{21}=r_{32}=r_{33}=0$), ϕ or ψ can be arbitrary (gimbal lock). In that case, we can adopt the convention ψ=0 and $\phi =\arctan\left(-r_{12},r_{22}\right)$.

Formally, the “mean rotation” is obtained using all *K* samples of $\phi_{i},\theta_{i},\psi_{i}$ in the following manner,

(12) $${}^{}{\hat{R}}_{}^{}=\mathrm{RPY}\left(\frac{1}{K}\sum_{i}^{K}\phi_{i},\frac{1}{K}\sum_{i}^{K}\theta_{i},\frac{1}{K}\sum_{i}^{K}\psi_{i}\right)$$

If individual transformations have some normalized degree of confidence $\mu_{i}$, where $\sum_{i}^{K}\mu_{i}=1$, then rotation and translation “averages” can be obtained in the classic way:

(13) $${}^{}{\hat{R}}_{}^{}=\mathrm{RPY}\left(\frac{1}{K}\sum_{i}^{K}\mu_{i}\phi_{i},\frac{1}{K}\sum_{i}^{K}\mu_{i}\theta_{i},\frac{1}{K}\sum_{i}^{K}\mu_{i}\psi_{i}\right)\;\mathrm{and}\;\hat{t}=\frac{1}{K}\sum_{i}^{K}\mu_{i}t_{i}$$

An even more compact and straightforward approach, even though with slightly different results (due to the least square fit technique associated), is to use SVD. First, we calculate the decomposition using traditional tools:

(14) $$\left[\begin{array}{ccc} U & \Sigma & V \end{array}\right]=\mathrm{SVD}\left(\sum_{i}R_{i}\right)$$

and then obtain the “average rotation” $\hat{R}$ using

(15) $${}^{}{\hat{R}}_{}^{}=VU^{⊺}.$$

In case different degrees of confidence in the geometric transformations are available, we can weight the various transformations ${}^{}R_{i}^{}$ using the following,

(16) $$\left[\begin{array}{ccc} U & \Sigma & V \end{array}\right]=\mathrm{SVD}\left(\sum_{i}\mu_{i}{}^{}R_{i}^{}\right),$$

assuming that $\sum_{i}^{N}\mu_{i}=1$, but not necessarily. Indeed, if a given ${}^{}R_{i}^{}$ has a larger confidence (for example the double of the remainder) it would be added twice in expression (16). In all cases, the final “average” transformation is given, of course, by ${}^{}{\hat{T}}_{}^{}=\left[\begin{array}{c} {}^{}{\hat{R}}_{}^{}\;|\;\hat{t} \end{array}\right]$.

## 4. Propagation of Uncertainty with a Simulated Experiment

**Figure 5:**
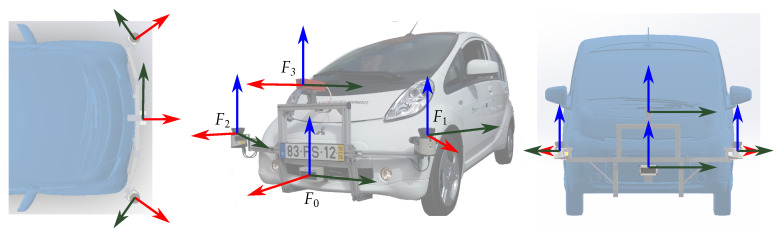
Example of four sensors in ATLASCAR2 vehicle.

To test the properties and potential advantages of the approach, we perform a simulation considering a set up based on the ATLASCAR2 prototype [35]. Figure 5 shows the car with four sensors placed on their own coordinate frames ($F_{0},F_{1},F_{2},F_{3}$). The geometric transformations between all pairs (${}^{0}{\tilde{T}}_{1}$, ${}^{0}{\tilde{T}}_{2}$, ${}^{0}{\tilde{T}}_{3}$, ${}^{1}{\tilde{T}}_{2}$, ${}^{1}{\tilde{T}}_{3}$, ${}^{2}{\tilde{T}}_{3}$) are measured experimentally, most likely with some errors both in translation and rotation.

This section will demonstrate with numerical examples and a systematic analysis that using combinations of multiple pairwise transformations, within certain conditions, decreases the error that occurs in each simple transformation between pairs of sensors in the car.

For this experiment, we use the real relative postures in terms of position (x,y,z), in meters, and Euler angles (ϕ,θ,ψ), in degrees, of the remainder three sensors, respectively, to the reference sensor ($F_{0}$), using the notation {x,y,z,ϕ,θ,ψ}, are defined as follows.

(17) $${}^{0}{\tilde{T}}_{1}^{}\to \left\{-0.05,-1,0.25,35,0,0\right\},{}^{0}{\tilde{T}}_{2}^{}\to \left\{-0.05,1,0.25,-35,0,0\right\},{}^{0}{\tilde{T}}_{3}^{}\to \left\{-0.02,0,0.50,0,0,0\right\}.$$

The approximate numerical values of the transformation matrices are then given by

(18) $${}^{0}{\tilde{T}}_{1}^{}=\left[\begin{array}{cccc} 0.8192 & -0.5736 & 0 & -0.05\\ 0.5736 & 0.8192 & 0 & -1\\ 0 & 0 & 1 & 0.25\\ 0 & 0 & 0 & 1 \end{array}\right],{}^{0}{\tilde{T}}_{2}^{}=\left[\begin{array}{cccc} 0.8192 & 0.5736 & 0 & -0.05\\ -0.5736 & 0.8192 & 0 & 1\\ 0 & 0 & 1 & 0.25\\ 0 & 0 & 0 & 1 \end{array}\right],{}^{0}{\tilde{T}}_{3}^{}=\left[\begin{array}{cccc} 1 & 0 & 0 & -0.02\\ 0 & 1 & 0 & 0\\ 0 & 0 & 1 & 0.05\\ 0 & 0 & 0 & 1 \end{array}\right]$$

We propose to determine ${}^{0}{\hat{T}}_{1}^{}$ by applying the algorithms described in the previous sections. As we have four sensors in the setup (N=4) we have five (see Table 1) possible transformation paths (19):

(19) $$\begin{array}{cc} {}^{0}{\hat{T}}_{1}^{0} & ={}^{0}{\tilde{T}}_{1}^{}\\ {}^{0}{\hat{T}}_{1}^{1} & ={}^{0}{\tilde{T}}_{2}^{}{}^{2}{\tilde{T}}_{1}^{}\\ {}^{0}{\hat{T}}_{1}^{2} & ={}^{0}{\tilde{T}}_{3}^{}{}^{3}{\tilde{T}}_{1}^{}\\ {}^{0}{\hat{T}}_{1}^{3} & ={}^{0}{\tilde{T}}_{2}^{}{}^{2}{\tilde{T}}_{3}^{}{}^{3}{\tilde{T}}_{1}^{}\\ {}^{0}{\hat{T}}_{1}^{4} & ={}^{0}{\tilde{T}}_{3}^{}{}^{3}{\tilde{T}}_{2}^{}{}^{2}{\tilde{T}}_{1}^{} \end{array}.$$

For the purpose of this experiment, we do not use any point clouds and we assume that the transformations are already available (for example, after using the matching technique mentioned earlier). To continue the experiment, we need all the multiple pairs of transformations ${}^{2}{\tilde{T}}_{1}^{}$, ${}^{3}{\tilde{T}}_{1}^{}$, ${}^{2}{\tilde{T}}_{3}^{}$, etc., which are calculated by simple algebraic manipulation: for example, ${}^{2}{\tilde{T}}_{1}^{}={\left({}^{0}{\tilde{T}}_{2}^{}\right)}^{-1}{}^{0}{\tilde{T}}_{1}^{}$, and so on. As it is expected, with these “perfect” geometric transformations, all the procedures presented earlier, namely, the average transformation given by expression (15) and related, produce the perfect result. In other words, the following operations,

(20) $$\left[\begin{array}{ccc} U & \Sigma & V \end{array}\right]=\mathrm{SVD}\left({}^{0}{\hat{R}}_{1}^{0}+{}^{0}{\hat{R}}_{1}^{1}+{}^{0}{\hat{R}}_{1}^{2}+{}^{0}{\hat{R}}_{1}^{3}+{}^{0}{\hat{R}}_{1}^{4}\right)\;\mathrm{with}\;{}^{0}{\hat{R}}_{1}^{}=UV^{⊺},$$

confirm that ${}^{0}{\hat{R}}_{1}^{}={}^{0}{\tilde{R}}_{1}^{}$, and the same for the translation part, which is easier to calculate (a simple arithmetic mean). However, the important issue here is to study the effect of errors in the transformations and how they propagate through the operations, and the calculation of the average transformation to reduce the error in the final ${}^{0}{\hat{T}}_{1}^{}$. For this purpose, a systematic test of uncertainty propagation was carried out using a Monte Carlo Simulation (MCS) to study the propagation of errors when multiplying the geometric transformations given in (19), and their subsequent combination.

The uncertainty in the transformation matrices may have origin in any of the the six variables: $\phi ,\theta ,\psi ,t_{x},t_{y},t_{z}$. Therefore, to apply the MCS method to study uncertainty propagation, an explicit expression for theses variables was derived for all matrix multiplications in (19) using expression (11) for the angles, and the three lines of the fourth column of the resulting matrix for the translation.

To perform this operation, the nominal values presented in (17) are used with an additional term that represents the uncertainty in each variable for each of the three base matrices, yielding a total of six sources of uncertainty for each matrix involved. In (21), the authors present an example of one of those transformation matrices with the six uncertainty values:

(21) $${}^{0}{\hat{T}}_{1}^{\Delta }=\left[\begin{array}{cccc} 1 & 0 & 0 & t_{x_{1}}+\Delta x_{1}\\ 0 & 1 & 0 & t_{y_{1}}+\Delta y_{1}\\ 0 & 0 & 1 & t_{z_{1}}+\Delta z_{1}\\ 0 & 0 & 0 & 1 \end{array}\right]\times \mathrm{RPY}\left(\phi_{1}+\Delta \phi_{1},\theta_{1}+\Delta \theta_{1},\psi_{1}+\Delta \psi_{1}\right).$$

For the the purpose of MCS, a new notation is introduced to identify a matrix that includes the uncertainty terms to study the uncertainty propagation: ${}^{0}{\hat{T}}_{2}^{\Delta }$.

However, to fully simulate uncertainty propagation in the expressions of (19), intermediate matrices, such as ${}^{2}{\tilde{T}}_{1}^{}$, ${}^{3}{\tilde{T}}_{1}^{}$, ${}^{2}{\tilde{T}}_{3}^{}$, etc., are also needed, but they are not available because in this simulation there are no point clouds to extract the matrices from them. Therefore, these matrices have to be derived from combinations of the others, but with their own terms of uncertainty and not the terms from the others that originate them by multiplication or inversion. This is necessary to have an unbiased experience and, of course, the consequence is to add more degrees of freedom for the MCS.

For example, ${}^{2}{\hat{T}}_{3}^{\Delta }$ can be obtained after ${\left({}^{0}{\hat{T}}_{2}^{\Delta }\right)}^{-1}\times {}^{0}{\hat{T}}_{3}^{\Delta }$ and if it were to be studied on its own (the propagation of uncertainty that reached it), no further manipulations would be required to study this uncertainty propagation. The issue is that ${}^{2}{\hat{T}}_{3}^{\Delta }$ is to be used in other expressions and for simulation purposes there should not exist unrelated terms of uncertainty with the same name. Therefore, for this example and all the others in a similar situation, the terms of uncertainty were renamed to allow true independence during the MCS tests. The procedure was to replace the $\left[\Delta \phi_{n},\Delta \theta_{n},\Delta \psi_{n}\Delta t_{x_{n}},\Delta t_{y_{n}},\Delta t_{z_{n}}\right]$ in ${}^{2}{\hat{T}}_{3}^{\Delta }$ by new terms, namely, in this specific case by $\left[\Delta \phi_{n32},\Delta \theta_{n32},\Delta \psi_{n32}\Delta t_{x_{n32}},\Delta t_{y_{n32}},\Delta t_{z_{n32}}\right]$.

For each of the matrix operations from (19), the MCS method was applied to the intricate analytic expressions resulting from the application of (11), where the variables depend on many sources of uncertainty: for example, matrix ${}^{0}{\hat{T}}_{1}^{4\Delta }$ depends on 30 sources of error from the accumulated operations in this simulation. This is actually a worst case scenario not usually occurring with experimental data.

Both uniform and Gaussian probability density functions (PDF) were used, and several uncertainty values (standard deviation) were tested for the angles and for the translation components. To ease the implementation of the process, in each trial, the same value in radians for angles and in meters for translations was used for all parameters. This option was taken for simple simulation convenience; therefore, an input uncertainty of 0.1 results in 0.1 rad, which is about 5.7°, and 0.1 m for the translation parts. The parameters of the full experiment are as follows.

**Figure 6:**
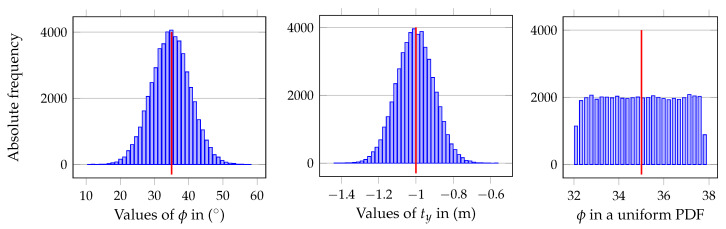
Histogram of variables ϕ and $t_{y}$, with mean value marked, and, respectively, $\sigma_{\phi }=4.8{}^{\circ}$ and $\sigma_{t_{y}}=0.08$ m when the original Gaussian uncertainty is 5.7° and 0.1 m. The third histogram on the right shows the final PDF of ϕ when the uncertainty is uniform, but now with $\sigma_{\phi }=1.36{}^{\circ}$.

- Number of samples for the MCS: $10^{5}$ for each transformation path;
- Standard deviations of input uncertainties:
  - for translations (in meter): {0.01, 0.02, 0.05, 0.1, 0.15, 0.2, 0.25, 0.3, 0.35, 0.4, 0.45, 0.5};
  - for angles: {0.6°, 1.1°, 2.9°, 5.7°, 8.6°, 11.5°, 14.3°, 17.2°, 20.1°, 22.9°, 25.8°, 28.6°};
- PDF for the samples: Gaussian and uniform distributions;

The final results are of the same nature for all six functions for the four transformation paths (five with the direct ${}^{0}{\hat{T}}_{1}^{\Delta }$). This means that the PDF of the uncertainties is preserved to the final output, namely, for the Gaussian PDF (Figure 6). Curiously, uniform PDF in the input maps also as uniform PDF for the variables in matrix ${}^{0}{\hat{T}}_{1}^{\Delta }$ (shown in the third histogram of Figure 6), but maps to Gaussian PDF for all the other (${}^{0}{\hat{T}}_{1}^{1\Delta },{}^{0}{\hat{T}}_{1}^{2\Delta }$, etc.).

The wealth of data generated by the MCS allows the analysis of the properties of this technique that is necessary for the multi-pairwise-based calibration. Two main issues must be observed: how the mean value of each variable is preserved among the several magnitudes of uncertainty, and how uncertainty (the standard deviation around the mean) is propagated on each transformation path. Finally, and following the central purpose of this multi-pairwise technique, what are the gains in terms or error propagation when combining (averaging) the results of all the transformation paths involved.

Table 2 shows the mean values of the six variables (orientations and translations) at the end of the five transformation paths, along with their mean values, including also the real ground truth values for easier comparison. This case was for an input uncertainty of 0.1 from a Gaussian PDF.

It can be seen that practically all transformation paths originate mean values very close to the real value in spite of all the uncertainties of the operations. Nonetheless, this performance degrades for higher values of uncertainty as detailed ahead.

The other important issue is the propagated uncertainty on each operation, and how is it compensated by the combination of the multiple results. As stated earlier, the standard deviation is the central measure and it is used to translate the uncertainty. When averaging multiple random variables, the standard deviation of the result ($\sigma_{M}$) is given by (22) where for this case five variables, and their individual standard deviations, are used:

(22) $$\sigma_{M}=\frac{\sqrt{\sigma_{0}^{2}+\sigma_{1}^{2}+\sigma_{2}^{2}+\sigma_{3}^{2}+\sigma_{4}^{2}}}{5}.$$

If $\sigma_{M}$ — the final mean propagated standard deviation — is smaller than the individual uncertainty of the simple pairwise transformation, then the process reduces the uncertainty present in that single geometric transformation. For the same example given in Table 2, Table 3 shows the standard deviation (propagated uncertainty) for each transformation path, along with the mean standard deviation ($\sigma_{M})$ that results from the average of the five transformation paths.

As Table 3 shows, except for $t_{x}$ and $t_{z}$ whose nominal values are too small when compared to the large input uncertainty, thus not relevant here, the combined propagated uncertainty is notoriously smaller than the initial uncertainty, even if the individual propagated uncertainty increases in the majority of the transformation paths. To better analyze and express the results, a concept named gain of propagated uncertainty, or $G_{\sigma }$, is created and defined as in (23),

(23) $$G_{\sigma }=\frac{\sigma_{I}-\sigma_{M}}{\sigma_{I}}=1-\frac{\sigma_{M}}{\sigma_{I}},$$

where $\sigma_{M}$ if the average propagated uncertainty and $\sigma_{I}$ is the initial uncertainty on the variables (0.1 m for translations or 5.7° for rotations in the previous example).

Table 4 shows more details on the results of the process taking as example one of the angles (ϕ) and one of the translations ($t_{y}$). This table covers results for several input uncertainties (named $\sigma_{R}$ for angles and $\sigma_{t}$ for translations) and, along with the mean values for the angle and the translation ($\bar{\phi }$, ${\bar{t}}_{y}$), shows both the final deviation of the variables ($\Delta_{r}\phi$ to express the relative deviation for angle ϕ, and $\Delta_{r}t_{y}$ for the relative deviation for translation $t_{y}$) and the gain in uncertainty reduction (labeled Error reduction in the table) showing the gains for ϕ and $t_{y}$ (respectively, $G_{\sigma_{\phi }}$ and $G_{\sigma_{t_{y}}}$).

From Table 4, we can conclude that the gains in uncertainty reduction can be as high as 17% for small uncertainties but reduce gradually when the input uncertainty reaches values beyond 10° or 0.2 m. Furthermore, the mean values of the variables almost do not degrade, although mean values of translation variables degrade faster than for angles for larger input errors.

**Figure 7:**
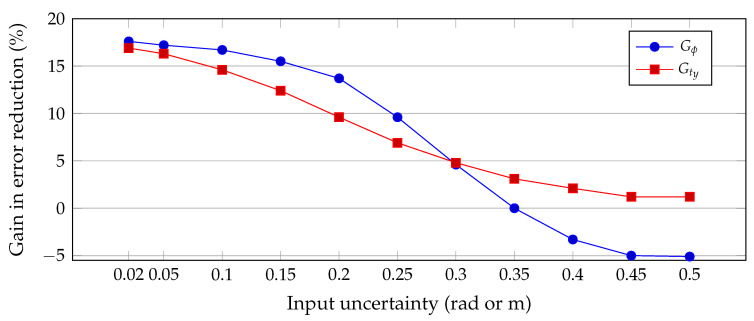
Gains in error reduction of the multi-pairwise approach for two variables: one rotation (ϕ) and one translation ($t_{y}$). For input uncertainties beyond 0.35 (radians or meters) the gain become loss, and the process is no longer advantageous, ceasing the usefulness of the technique for this setup.

Figure 7 plots the gains in uncertainty reduction for the two variables analyzed in detail, and we can even observe the case where the gains become negative for input uncertainties after the value 0.35 (rad for the angles), making the approach theoretically ineffective in terms of uncertainty reduction. Nonetheless, note that the mean values of variables (at least those with nominal values much larger than the input uncertainty) still hold close to the real value.

In summary, as long as mean values of variables do not deviate too much and the error reduction is positive and meaningful (possibly around 10% or more), the technique of multi-pairwise combinations of geometric transformations is valid and useful.

This MCS testing methodology has shown great reliability because different repetitions yielded these same tables of results with occasional fluctuations only in some decimal places, despite the random nature of the MCS. Moreover, the tests were performed with other nominal values besides those presented in expression (17) and, as would be expected due to the nature of MCS, the results and limits of the propagated errors are similar for the same number of geometric transformations.

For complementary comparison, Table 5 shows something similar to Table 4, but only with transformation paths up to length 2; that is, only three transformation paths to combine.

It can be observed that the results on Table 5, where only three transformation paths are used, and not five as earlier, are poorer, especially in the error reduction gain. From these two last tables it can be concluded that the most interesting solution is to combine more transformation paths, but shorter transformation paths are preferable because they propagate less uncertainty (as Table 3 confirms).

The previous observation can be used to corroborate one of the heuristic proposals made earlier on how to pick which transformation paths to combine. Therefore, a good proposal seems to be use more short transformation paths instead of fewer longer transformation paths. For many sensors this would require a further study to find the best trade-off of on many transformation paths of length 3, four or more would be useful to add to the list of all transformation paths of length 2.

To conclude this analysis, a last table (Table 6) is presented where the PDF of the input uncertainties is taken as uniform and not Gaussian. Here, five transformation paths were used for the same values of initial uncertainties as before. The most noticeable observation is the large gain in uncertainty reduction (more than 75%) for all tested uncertainties. Indeed, uniform distributions keep the error limited and restrict the propagation. Mean values are well preserved for all variables and standard deviations kept low (though still larger for translations) but, in the end, showing clear benefits of using the multi-pairwise technique. The only caveat is that, most likely, the sources of error cannot be considered uniform, that is, not strictly limited to an interval, and that is why Gaussian approaches, being more conservative, appear thus to be safer in performing uncertainty predictions in this context.

In conclusion, this section demonstrated the viability and advantage of the multi-pairwise technique to perform sensor calibration within some conditions and limits. For a set-up with four sensors, the technique is advantageous and viable if initial angular uncertainties are less than about 15° and translation uncertainties less than about 0.15 m. If these restrictions are ensured, then, statistically, and assuming Gaussian uncertainties, the multi-pairwise approach improves the accuracy of the calibration among multiple sensors.

In summary, the operation of “averaging” geometric transformations can reduce the error present in the relative position of sensors. Nonetheless, although demanding and perhaps too conservative, the results of this theoretical approach are harder to compare with those from experiments using real data because ground truth is usually not available or not very precise. Therefore, in real data experiments, either there is an estimate of the ground truth of the sensor relative placements, or some alternative metric has to be used, such as the mean square deviations of 3D points recalculated with the estimated geometric transformations. The next section, dedicated to results, describes these issues in detail.

## 5. Results

The previous section presented an analysis of uncertainty propagation using a case study for more clarity, but demonstrated that, in some conditions, the multi-pairwise approach reduces the uncertainty in calculating the geometric transformations, by analyzing the propagated uncertainty in final orientations and translations of the several sensors relative localization, which is the essence of extrinsic calibration.

This section provides an approach using sets of points (also named point clouds throughout this paper) to extract the single pairwise transformations and obtain the multi-pairwise transformation and compare performance in two perspectives.

Indeed, this procedure to obtain results hinders a subtle, but determinant, change as the geometric transformations do not exist as before in Section 4, but are extracted after the point clouds, which will lead to interesting results as discussed further. Experiments were done both with real data from sensors and simulated point clouds.

The experiment used four different sensors. The principle is independent of the nature of the sensor, as long as it can detect the center of the ball, so four different types of cameras were used: RGB, monochromatic, infrared, and Kinect-based (for depth maps).

An example of simple pairwise-based calibration has been previously used for the first three cameras [8]. In the current work, we add a depth sensor to enrich the initial set-up. Indeed, depth maps are obtained and, being 2D images, ball positions are detected with a Deep Learning trained network used for RGB, mono, and IR images, which are all native, or converted to, simple grayscale intensity images.

**Figure 8:**
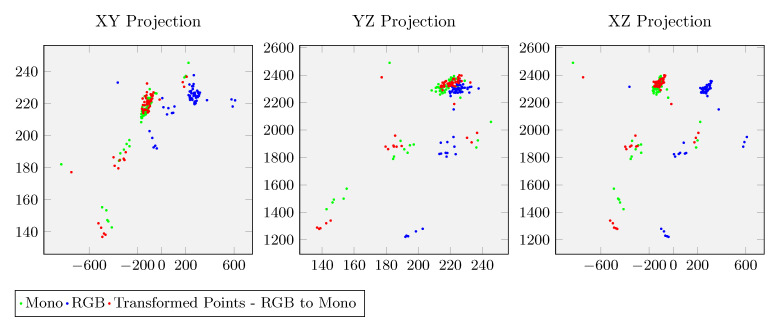
Illustration of the results of calibrating a RGB and Mono camera. Through the registration procedure, green points are transformed into red points which match closely the blue point cloud.

The detection of the ball center on all sensors is performed using Detectron2 deep learning techniques [36] to detect a ball in successive frames. Figure 8 shows an example for the calibration procedure with three point clouds: one obtained from the RGB camera (blue), another from the Mono camera (green), and the third (red), which is the calibrated version of the green relatively to the RGB camera.

In the experiments carried out, the four cameras were placed in several positions ensuring a common field of view favorable to capture the ball during its motion. In each frame, the center of the ball is detected and saved in a point cloud of all the viewed ball centers for each of the four cameras. This center can be calculated in the coordinate frame of the camera, in meters, using optics and lenses equations (intrinsic matrix). By using the methods presented earlier for matching point clouds, the pairwise transformations between the sensors are estimated, opening the way to the application of the multi pairwise method proposed in this paper.

Contrarily to simulation, no ground truth is available for the experimental data. Therefore, the metrics to assess the performance of the method is based on the deviation between the point cloud from the reference sensor and the point cloud from another sensor after the calibration process. For the metric based on distance deviations, we use the relative deviation (in percentage) of the root mean square (RMS) of absolute errors which are measured in mm.

Experiments have shown that outliers occur frequently in the ball detection phase. That can have several reasons and depends on the type of sensor used. The deep learning detection algorithm shows most of the times very high levels of confidence (98%), but sometimes they are lower. The resolution of some cameras (namely, the infrared) is much lower than the other sensors, creating uncertainties in obtaining the center point of the ball; for some sensors, it is harder to obtain precise intrinsic parameters and, again, the infrared camera is the strongest example. Additionally, illumination conditions are not always ideal and that too can affect precise detection. All these issues may occur in smaller or larger extents and can generate errors in the ball center detection and outliers.

**Figure 9:**
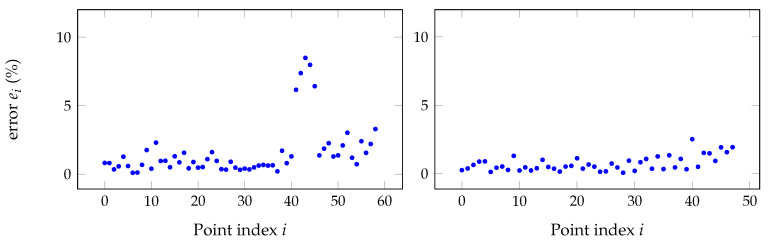
Percentage of error between each point of one point cloud with another point cloud before (**left**) and after (**right**) the application of Chauvenet criterion for outliers removal.

These outliers can be detected by evaluating the distance between the reference point cloud and the transformed point clouds of the sensors (using the pairwise transformation). Figure 9 (left) illustrates the situation for the case of two sensors where several samples (after sample number 40) exhibit a large disparity when compared to the remainder. The error displayed in the plot is calculated for each point $P_{i}$ of point cloud from sensor 0 (reference) to respective point $Q_{i}$ of point cloud from sensor *m*, after calibration, in the following way.

(24) $$e_{i}=\frac{\left|\right|P_{i}-{}^{0}{\hat{T}}_{m}^{}Q_{i}\left|\right|}{\left|\right|P_{i}\left|\right|}$$

Clearly, some of the points illustrated are outliers and result possibly from a poor detection of the target in one, or both, sensors. Those original samples that do not match a given criteria have to be removed from both point clouds to keep the correspondence for the pairwise calculation.

The process selected to eliminate outliers is the Chauvenet criterion, first devised by William Chauvenet in 1863 [37], but broadly applied and documented in many sources [38]. This technique identifies samples that fail to fit a normal distribution with a given probability based on their deviation from the mean using also the standard deviation for the calculation. The samples that fail the test are considered outliers, and the pairwise calculation of transformations is recomputed with the “clean” set of samples.

The Chauvenet test can be applied recursively until no outliers remain. However, if the distribution is not Gaussian (or unknown), the process may exaggerate the pruning of the data set and can remove too many samples. Therefore, in this work, two iterations of the criterion are applied to ensure that potential residual outliers from the first iteration are removed. With the point clouds freed from the outliers (as in the right side of Figure 9), the calculation of calibration matrices, both single- and multi-pairwise, can be performed with more confidence.

**Figure 10:**
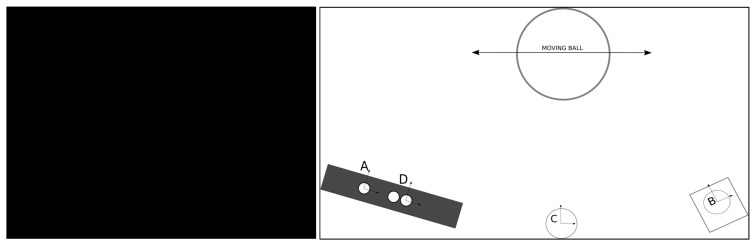
Example of set-up to collect data with the sensors placed in variate positions and orientations. A is the depth camera, B the IR camera, C the monochromatic camera, and D the RGB camera.

To test and analyze the technique proposed, simple experimental set-ups with the four sensors were used as the one illustrated in Figure 10.

**Figure 11:**
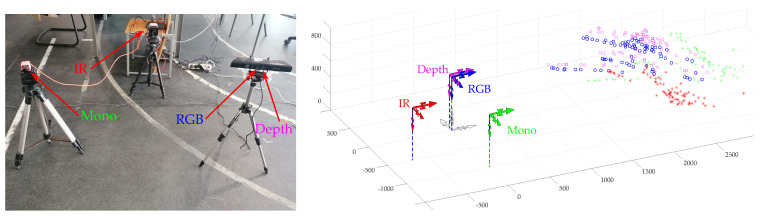
Example of another setup to collect data from sensors. On the right an actual collection of point cloud that shows serious defects making them mostly unusable for calibration. Units are in mm.

Similarly to the set-up in Figure 10, other data collections were gathered using other set-ups and sensor arrangements. For example, the set-up shown in Figure 11 generated the point clouds illustrated on the right, where clearly some of them are defective, compromising the proper illustration of the concepts being proposed in this paper. This may have occurred both for the reasons described earlier and for other unexpected factors that restricted the data acquisition procedures.

To overcome the restrictions encountered in the particular experimental data collection, as the one shown in Figure 11, it was also decided to synthesize point clouds on a similar configuration and add noise in all coordinates as an attempt to emulate real point clouds. Although these points and the results thereof are not from real sensors, they hopefully follow the pattern of a real setup, and the noise emulates some of the acquisition errors. The results were analyzed using two different metrics: one based on the relative value of the root mean square of the distance between point clouds (from the reference sensor and from the calibrated sensor), as given by expression (24), and a second one based on the deviation of the calibration matrices, in a line similar to the study performed in Section 4, and deviation in translations and Euler angles are assessed. The first metric can be applied without having any ground truth (compare the results with single versus multi pairwise) and the second one requires a ground truth to compare the calibration matrices from single- and multi-pairwise approaches with the correct value. This second metric can be used in simulated data only because only there a ground truth is available.

**Figure 12:**
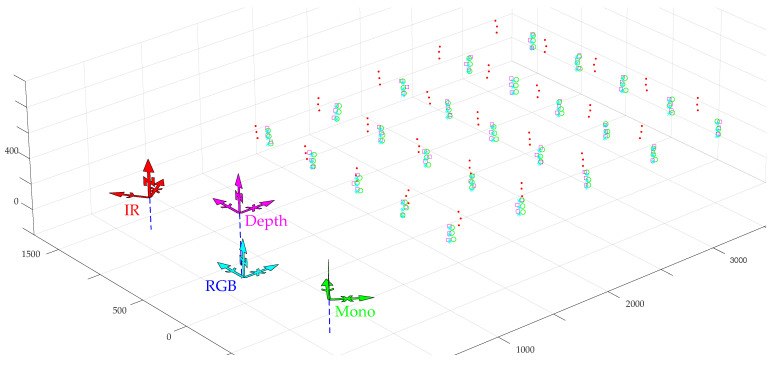
Example of synthetic point clouds with systematic and random errors in a sensor arrangement similar to the one in ATLASCAR2. Units are in mm.

Multiple experiments were done with simulated point clouds, both encompassing systematic and random errors as the one shown in Figure 12 where nominal points follow a grid-like layout for easier visual tracking, but other arrangements, even fully random, were tested.

**Figure 13:**
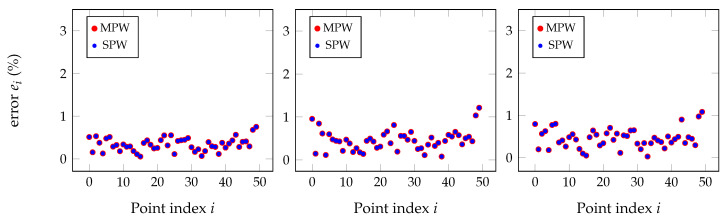
Relative distance errors between every pair of points for the SPW and MPW approaches after removing the outliers with the Chauvenet method for an input error of about 10 mm in the point clouds. Both techniques give practically the same results, as shown by the clear overlap of plots.

However, almost all results have shown little or no improvement at all of the direct application of the multi-pairwise (MPW) matrices when compared to the single-pairwise (SPW). Figure 13 shows the detailed point-to-point relative error for the pairs mono to depth, mono to RGB, and mono to IR for some other simulated point clouds, and differences between methods are barely noticeable.

An explanation for what was observed in the simulated data is given next. In ideal conditions, the SPW and MPW approaches give the same results for multisensor calibration, making it unnecessary to use the later. Additionally, as verified, when using synthetic point clouds, the advantages of MPW have shown to be too little to be useful. Indeed, when comparing with the study made in Section 4, now the geometric transformations are not all truly independent since they are obtained after pairs of point clouds; therefore, the same point cloud is used multiple times for different geometric transformations. These effects are even stronger in simulated data, and that is why the experiments were redirected into another point of view. The validity of transformations using MPW still stands but, as it is shown next, it will be used in a indirect approach.

**Figure 14:**
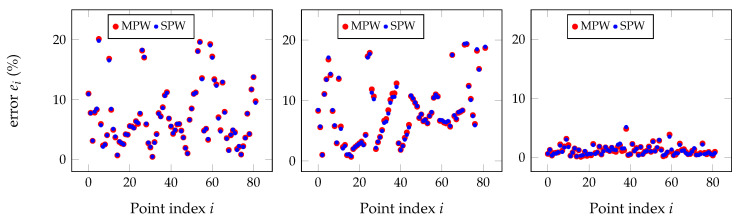
Root mean square (RMS) errors for SPW and MPW for the three sensors relatively to the reference sensor (RGB camera) for the raw point clouds with 82 points obtained in the setup from Figure 10.

**Figure 15:**
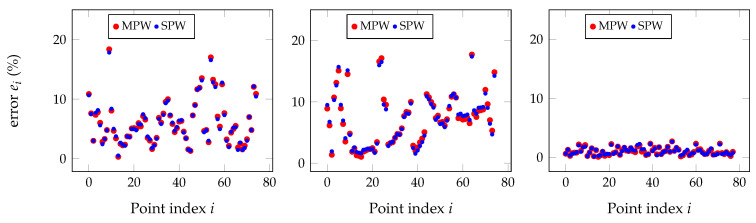
RMS errors for SPW and MPW for the three sensors relative to the reference sensor for the point clouds whose results are shown in Figure 14 but where outliers have been removed by Chauvenet criterion. Number of removed outliers varies with the combination of sensors.

As the simulated point clouds were not as rich as real data, the experiment presented earlier from the setup in Figure 10 was chosen and analyzed from another perspective. Both SPW and MPW approaches were tried, as shown in Figure 14 and also in the Chauvenet polished version in Figure 15, and there are points in the real point clouds for which the MPW performs better than for the SPW, and vice versa.

In average, for the entire point cloud, the MPW performs similarly to the SPW, but individually in each point it performs either better or worse, which is a hint to exploit further.

Figure 15 shows the individual point errors after applying the Chauvenet criterion to eliminate outliers on the raw point clouds with the results shown in Figure 14.

After observing the results in Figure 14 or Figure 15, the hypothesis proposed is that poorer performance in the MPW in some points can represent situations of points that were captured with larger errors and compromised the point clouds and the geometric transformation generated after them. Therefore, using the real data, a series of experiments was carried out in order to improve the quality of the point clouds using the MPW approach. The workflow is the following for four sensors.

- Obtain the three SPW transformations after the four point clouds.
- Obtain the associated MPW transformations using all SPW and all the other inter-point cloud transformations.
- Apply the Chauvenet criterion to eliminate points by analyzing the recalculated error using the SPW with expression (24).
- Recalculate SPW and MPW matrices for the three sensors relatively to the reference sensor.
- Eliminate from the point clouds all points that have a larger error when using the MPW matrix.
- With the new filtered point cloud, recalculate the SPW matrix and use it as final value for the geometric transformation w.r.t. the reference sensor.

**Figure 16:**
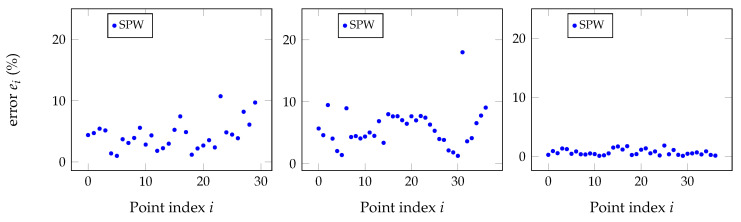
RMS errors for the three sensors relative to the reference sensor for the point clouds whose results are shown in Figure 15 but with additional points removed by observing the MPW versus the SPW results. The observable conclusion when comparing to previous figures is that additional outliers have been removed even after the application of the Chauvenet criterion.

The procedure just described was applied and produced the plots of Figure 16, and the results are summarized in Table 7.

From Table 7, it is clear that the process of removing outliers decreases the RMS error along with its standard deviation, which is equivalent to state that the geometric transformations (calibration matrices) are more accurate than the ones calculated with the raw point clouds. The reduction of also the standard deviation reinforces the fact that more accurate point clouds are obtained in this process.

The Chauvenet criterion gives a first step in discarding outliers, but the analysis of the MPW for each point allows the elimination of additional selected points, thus giving smaller mean errors. In the end, as shown in the last column of Table 7 there is a substantial reduction of the RMS error associated to the calibration matrix for each sensor relatively to the reference sensor. In the particular case of sensor 3, the mean error was already small because sensor 3 and the reference sensor lay attached on the same physical structure, but even there occurs an improvement on the point cloud, and therefore a better calibration matrix is obtained.

The results just presented demonstrate the usefulness of the MPW technique to assist the improvement of the point clouds in order to obtain better transformation matrices for the multi sensor extrinsic calibration process.

## 6. Conclusions

This paper proposes a methodology to perform semiautomatic calibration of several sensors. The requirement is to detect, simultaneously in all the sensors, corresponding 3D points in space, for example, by detecting the center of a moving ball in their field of view. The solution builds upon a technique proposed in previous works [1,8], by combining multiple geometric transformations in several transformation paths to refine or complement the computation of extrinsic parameters of all sensors w.r.t. one sensor defined as the reference. We refer to this approach as a multi pairwise approach for sensor extrinsic calibration, as, instead of considering only transformations between pairs of sensors (as is the case of many previous works), this technique takes into account all sensors to establish the geometric relation between each pair. The technique also proposes alternatives to limit the number of transformation paths to use in order to circumvent the potential explosion in the number of paths that can originate from a transformation graph.

The technique has some advantages and applications, although not all of them always occurring, depending on the data and nature of sensors. Indeed, there are two different ways of using the MPW. First, if estimations of all geometric transformations between pairs of sensors exist and are obtained independently of each other, even if under some conditions, the MPW gives a solution with less error than SPW as was demonstrated in Section 4. On the other hand, if point clouds are available instead and geometric transformations are obtained using matching techniques, the MPW technique allows a refinement of the point clouds by eliminating points that escaped traditional outlier filtering, as demonstrated in the experimental results using the Chauvenet criterion.

More specifically, the technique was demonstrated theoretically to reduce the propagation of uncertainty under certain conditions. For a configuration inspired on a real setup with an instrumented car, initial uncertainty of values of up to 0.3 rad or 0.3 m on all angles and translations, are reduced by the MPW approach, which shows its advantage, as was demonstrated with a Monte Carlo Simulation methodology.

When point clouds are available, as shown in the results section, and that is probably the most common situation in the context of this work, the geometric transformations are not all independent and the simple replacement of the SPW matrix by the MPW may not result in error reduction. However, the MPW results in individual points can be used to filter out points that exhibit errors that degrade the calculation of the SPW (and the MPW all along). The outlier removal by Chauvenet criterion produces interesting results, but the further elimination of points with the application of the MPW refined the point clouds, allowing a new geometric transformation with even less error.

It was also verified that the uncertainty on the calculated calibration matrix is better for a larger number of transformation paths, and preferably using the shortest paths. Indeed, shorter transformations paths propagate less uncertainty and more transformation paths reduce the final uncertainty. In the cases presented using four sensors, five transformation paths exist, one with length 1 (the direct path, the one used for the SPW), two with length 2, and two with length 3. In the context of the approach described in Section 4, using all five transformation paths achieves better results than using only three of them. This effect would be stronger in set-ups with more sensors and more transformation paths. This opens a door for future developments by analyzing the several transformations paths available and pick only some of them that fit some criteria to be investigated.

The paper focused mainly on the advantages of the multi pairwise approach in two different perspectives and contexts of the available data, but the overall technique using the ball can be developed further. Indeed, the technique presented does not yet solve all the challenges of multimodal extrinsic calibration. An opportunity remains to merge with optimization techniques discussed in the related work. Other future developments will continue with the purpose of overcoming limitations inherited from the earlier approach, and not yet tackled in the present work, like the ambiguity in the detection of the ball hemisphere which occurs in some LIDAR sensors. Solutions for those challenges will possibly count with 3D dynamic tracking of the ball put in motion using more elaborate motion patterns.

## Figures and Tables

**Table 1:** Number of paths of the several lengths of the transformation paths for a given number of sensors *N*, and the full total of transformation paths in each set of sensors. The full total in each row is obtained by $\left(N-1\right)\times \sum_{r}L_{r}\left(T\right)$ or similarly, $\left(N-1\right)\times Q_{N}$ with $Q_{N}$ obtained from (3).

*N*	Lengths of Transformation Paths L(T) for Each Sensor in the Set									Full Total
	1	2	3	4	5	6	7	8	9	
3	1	1								4
4	1	2	2							15
5	1	3	6	6						64
6	1	4	12	24	24					325
7	1	5	20	60	120	120				1956
8	1	6	30	120	360	720	720			13,699
9	1	7	42	210	840	2520	5040	5040		109,600
10	1	8	56	336	1680	6720	20,160	40,320	40,320	986,409

**Table 2:** Mean values of the six variables for the transformation paths using a Gaussian distribution of errors on all variables with an initial uncertainty of 5.7° for angles and 0.1 m for translations.

	Real Value	${}^{0}{\hat{T}}_{1}^{}$	${}^{0}{\hat{T}}_{1}^{1}$	${}^{0}{\hat{T}}_{1}^{2}$	${}^{0}{\hat{T}}_{1}^{3}$	${}^{0}{\hat{T}}_{1}^{4}$	Overall Mean
$\bar{\phi }$ (${}^{\circ }$)	35	35.02	34.96	35.03	34.99	35.03	35.01
$\bar{\theta }$ (${}^{\circ }$)	0	0.01	0.03	0.04	−0.08	−0.03	−0.01
$\bar{\psi }$ (${}^{\circ }$)	0	−0.02	0.05	−0.03	−0.02	0.04	0.00
${\bar{t}}_{x}$ (m)	−0.05	−0.05	−0.05	−0.05	−0.05	−0.05	−0.05
${\bar{t}}_{y}$ (m)	−1	−1.00	−0.96	−0.98	−0.94	−0.94	−0.96
${\bar{t}}_{z}$ (m)	0.25	0.25	0.25	0.24	0.25	0.25	0.25

**Table 3:** Standard deviations of the several posture variables for the transformation paths assuming a Gaussian distribution of errors on all variables with an initial uncertainty of 5.7° or 0.1 m.

	${}^{0}{\hat{T}}_{1}^{}$	${}^{0}{\hat{T}}_{1}^{1}$	${}^{0}{\hat{T}}_{1}^{2}$	${}^{0}{\hat{T}}_{1}^{3}$	${}^{0}{\hat{T}}_{1}^{4}$	$\sigma_{M}$
$\sigma_{\phi }$ (${}^{\circ }$)	5.723	10.031	9.985	12.978	12.952	4.7718
$\sigma_{\theta }$ (${}^{\circ }$)	5.726	9.845	9.822	12.674	12.657	4.6777
$\sigma_{\psi }$ (${}^{\circ }$)	5.734	10.002	10.006	12.968	12.939	4.7692
$\sigma_{t_{x}}$ (m)	0.100	0.328	0.223	0.382	0.380	0.1353
$\sigma_{t_{y}}$ (m)	0.100	0.178	0.177	0.232	0.236	0.0854
$\sigma_{t_{z}}$ (m)	0.100	0.328	0.223	0.381	0.380	0.1351

**Table 4:** Summary of uncertainty propagation when using 5 paths with Gaussian PDF for input uncertainties in the MCS analysis. Example for two variables ϕ and $t_{y}$. The last two columns in the table show the gains in error reduction by using the multi pairwise approach.

Input Uncertainty		Results for ϕ and $t_{y}$						Error Reduction	
$\sigma_{R}{(}^{\circ })$	$\sigma_{t}\left(m\right)$	$\bar{\phi }{(}^{\circ })$	$\Delta_{r}\phi$	${\bar{t}}_{y}\left(m\right)$	$\Delta_{r}t_{y}$	$\sigma_{\phi }{(}^{\circ })$	$\sigma_{t_{y}}\left(m\right)$	$G_{\sigma_{\phi }}$	$G_{\sigma_{t_{y}}}$
0.6	0.01	35.00	0.0%	−1.00	0%	0.47	0.01	17.5%	16.9%
1.1	0.02	35.00	0.0%	−1.00	0%	0.95	0.02	17.5%	16.9%
2.9	0.05	35.01	0.0%	−0.99	1%	2.37	0.04	17.4%	16.4%
5.7	0.1	34.99	0.0%	−0.96	4%	4.77	0.09	16.8%	14.8%
8.6	0.15	34.98	0.1%	−0.92	8%	7.24	0.13	15.8%	12.4%
11.5	0.2	35.00	0.0%	−0.86	16%	9.92	0.18	13.5%	9.5%
14.3	0.25	34.93	0.2%	−0.80	26%	12.95	0.23	9.6%	7.0%
17.2	0.3	34.54	1.3%	−0.72	39%	16.39	0.29	4.7%	4.8%

**Table 5:** Summary of uncertainty propagation when using 3 paths with Gaussian PDF for input uncertainties. The last two columns in the table show the gains in error reduction by using the multi pairwise approach. The results are poorer than those shown in Table 4.

Input Uncertainty		Results for ϕ and $t_{y}$						Error Reduction	
$\sigma_{R}{(}^{\circ })$	$\sigma_{t}\left(m\right)$	$\bar{\phi }{(}^{\circ })$	$\Delta_{r}\phi$	${\bar{t}}_{y}\left(m\right)$	$\Delta_{r}t_{y}$	$\sigma_{\phi }{(}^{\circ })$	$\sigma_{t_{y}}\left(m\right)$	$G_{\sigma_{\phi }}$	$G_{\sigma_{t_{y}}}$
0.6	0.01	35.00	0.0%	−1.00	0.0%	0.5065	0.0089	11.6%	11.2%
1.1	0.02	35.00	0.0%	−1.00	0.1%	1.0117	0.0177	11.7%	11.3%
2.9	0.05	35.00	0.0%	−1.00	0.5%	2.5270	0.0446	11.8%	10.8%
5.7	0.1	35.00	0.0%	−0.98	2.0%	5.0754	0.0899	11.4%	10.1%
8.6	0.15	34.97	0.1%	−0.96	4.6%	7.6610	0.1366	10.9%	8.9%
11.5	0.2	34.99	0.0%	−0.92	8.3%	10.3880	0.1859	9.3%	7.1%
14.3	0.25	35.09	0.3%	−0.88	13.4%	13.2510	0.2367	7.5%	5.3%
17.2	0.3	34.97	0.1%	−0.84	19.6%	16.4970	0.2898	4.0%	3.4%

**Table 6:** Summary of uncertainty propagation when using five paths with uniform PDF for input uncertainties in the MCS analysis. The last columns in the table show the gains in error reduction by using the multi pairwise approach.

Input Uncertainty		Results for ϕ and $t_{y}$						Error Reduction	
$\sigma_{R}{(}^{\circ })$	$\sigma_{t}\left(m\right)$	$\bar{\phi }{(}^{\circ })$	$\Delta_{r}\phi$	${\bar{t}}_{y}\left(m\right)$	$\Delta_{r}t_{y}$	$\sigma_{\phi }{(}^{\circ })$	$\sigma_{t_{y}}\left(m\right)$	$G_{\sigma_{\phi }}$	$G_{\sigma_{t_{y}}}$
0.6	0.01	35.00	0.00%	−1.00	0.01%	0.137	0.002	76.2%	76.1%
1.1	0.02	35.00	0.00%	−1.00	0.01%	0.273	0.005	76.2%	76.0%
2.9	0.05	35.00	0.00%	−1.00	0.08%	0.682	0.012	76.2%	76.0%
5.7	0.1	35.00	0.00%	−1.00	0.30%	1.363	0.024	76.2%	76.0%
8.6	0.15	34.99	0.02%	−0.99	0.69%	2.047	0.036	76.2%	75.9%
11.5	0.2	35.00	0.00%	−0.99	1.21%	2.739	0.048	76.1%	75.8%
14.3	0.25	35.01	0.03%	−0.98	1.92%	3.429	0.061	76.1%	75.7%
17.2	0.3	34.98	0.07%	−0.97	2.77%	4.123	0.073	76.0%	75.6%
20.1	0.35	35.00	0.01%	−0.96	3.76%	4.814	0.086	76.0%	75.5%
22.9	0.4	35.00	0.00%	−0.95	4.94%	5.528	0.099	75.9%	75.3%
25.8	0.45	34.99	0.03%	−0.94	6.31%	6.230	0.112	75.8%	75.2%
28.6	0.5	35.03	0.07%	−0.93	7.89%	6.976	0.125	75.7%	75.0%

**Table 7:** Summary of results for the RMS relative error and its associated standard deviation between each sensor and the reference sensor upon outlier removal with Chauvenet criterion and using MPW. Errors are in percent values as well as their respective standard deviations (stdev). The last column shows the reduction factor (final value/initial value) of mean errors and standard deviations from original raw point cloud to the final point cloud purged from outliers. Overall, mean errors and standard deviations for SPW and MPW are similar so only one of them is shown.

Target Sensor		Raw Point Cloud	Chauvenet Removal	MPW Based Removal	Reduction Factors
1	Total points	82	75	29	
	Mean error	7.0	5.4	4.3	61.4%
	Stdev	4.9	3.6	2.3	46.9%
2	Total points	82	75	36	
	Mean error	8.5	6.5	5.8	68.2%
	Stdev	6.0	4.0	3.0	50.0%
3	Total points	82	78	36	
	Mean error	1.2	1.0	0.7	58.3%
	Stdev	0.8	0.6	0.5	62.5%
